# Multiple cardiovascular complications of COVID-19 infection in a young patient: a case report

**DOI:** 10.11604/pamj.2021.38.192.27471

**Published:** 2021-02-19

**Authors:** Hammam Rasras, Abdelaziz Boudihi, Anass Hbali, Nabila Ismaili, Noha El Ouafi

**Affiliations:** 1 Department of Cardiology, Mohammed VI University Hospital of Oujda, Mohammed First University of Oujda, Oujda, Morocco,; 2 Laboratory of Epidemiology, Clinical Research and Public Health, Faculty of Medicine and Pharmacy, Mohammed the First University of Oujda, Oujda, Morocco

**Keywords:** COVID-19, myocarditis, thrombosis, cardiogenic shock, case report

## Abstract

COVID-19 infection is responsible for many complications, which can lead to a high risk of mortality in some patients. Among them are cardiovascular complications which are classified as the most severe. We report a case of a young woman, with no relevant pathological history, admitted for COVID-19 infection, complicated by myocarditis with severe ventricular dysfunction, cardiogenic shock and a large thrombosis into the left ventricle (LV) that was responsible for a left lower limb ischemia associated with a deep venous thrombosis of right lower limb.

## Introduction

Since the beginning of the COVID-19 pandemic, many studies were carried out for describing its characteristics, complications, as well as best therapeutic approaches. Among these complications, come cardiac impairments, especially, myocardial injury and arteriovenous thrombosis, which are quickly emerged as a major medical challenge despite a well-conducted preventive anticoagulation. They are presented with a high incidence, especially with critical patients, also with a high mortality rate that accompanies them [1].

Here, we report a case of a young woman who presented multiple cardiovascular complications of COVID-19. Through this case, we aim to describe the severity of this infection, as well as therapeutic approaches suggested so far for the management of these complications.

## Patient and observation

This is the case of a 47-year-old woman with no relevant medical history. Twenty days before admission, the patient had a progressively worsening dyspnea preceded by cough and fever, which were initially neglected. On admission, the patient was asthenic with severe dyspnoea and pain in both lower limbs. On clinical examination, she was hypothermic (35.1°), presented with cardiogenic shock (blood pressure (BP) at 80/45 mmHg, heart beats (HB) at 150, oliguria and mottling in both lower limbs), spontaneous SpO2 was 80%, HOMANS sign was positive in the right lower limb with ischemic signs in the left lower limb (pain, hypoesthesia and absence of pulse).

**Figure 1:**
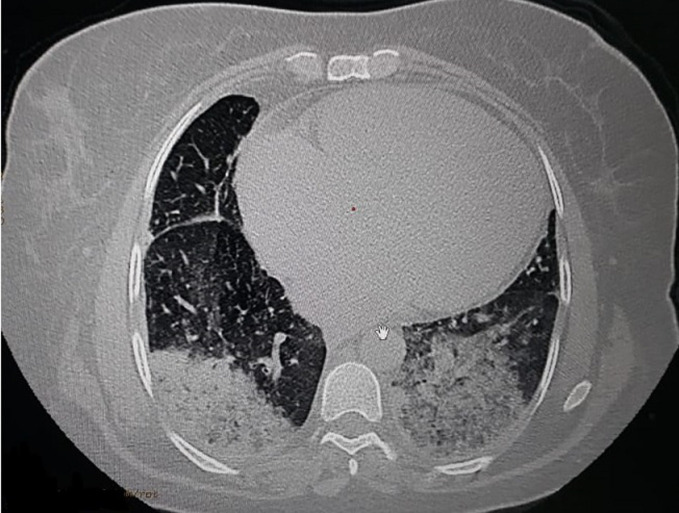
thoracic CT scan showing diffuse patchy ground-glass opacities suggesting COVID-19 pneumonia

In the para clinical work up, a biological inflammatory syndrome (White Blood Cells: 20,000/mm^3^, CRP: 147 mg/l and a procalcitonin at 2.9 mg/l, fibrinogen: 8.7 g/l, lactate dehydrogenase: 1542 g/mol with ferretinemia: 2150 mg/l) was found, and because of the pandemic context, a thoracic computed tomography (CT)- scan was performed; showing diffuse patchy ground - glass like opacities with multiple areas of condensation suggesting COVID-19 pneumonia (Figure 1), a COVID-19 polymerase chain reaction (PCR) test was realized and was positive, with D-dimers: 3.40 μg/l.

**Figure 2:**
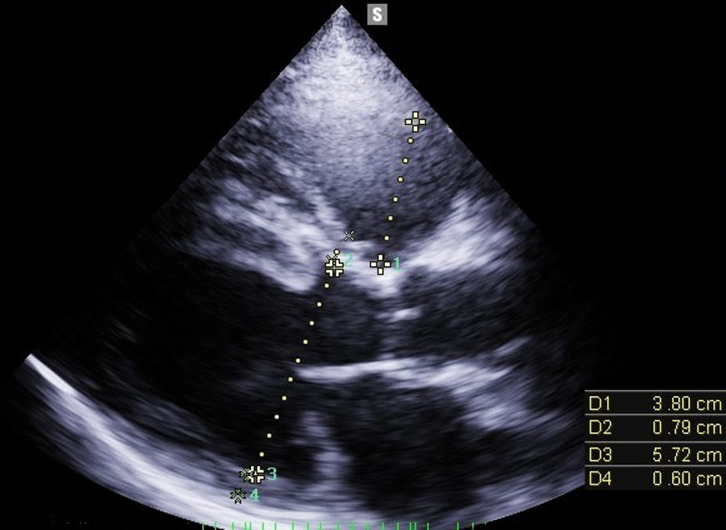
para sternal long axis view of TTE that shows bi-ventricular dilatation (tele diastolic diameter of LV 57 mm with that of the RV 38 mm)

**Figure 3:**
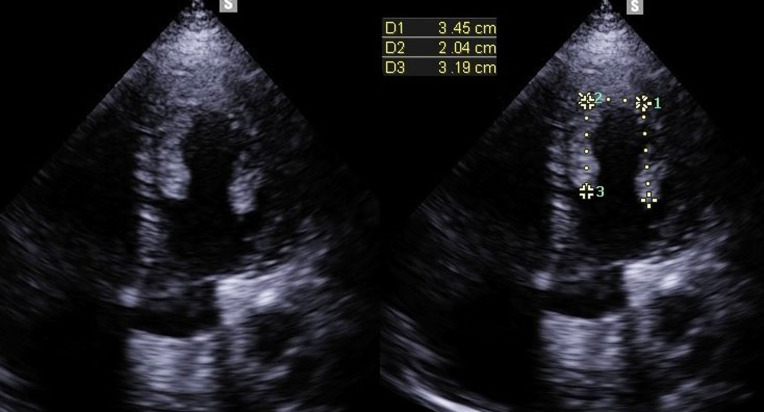
apical view of TTE that shows a large intra LV horseshoe thrombus

**Figure 4:**
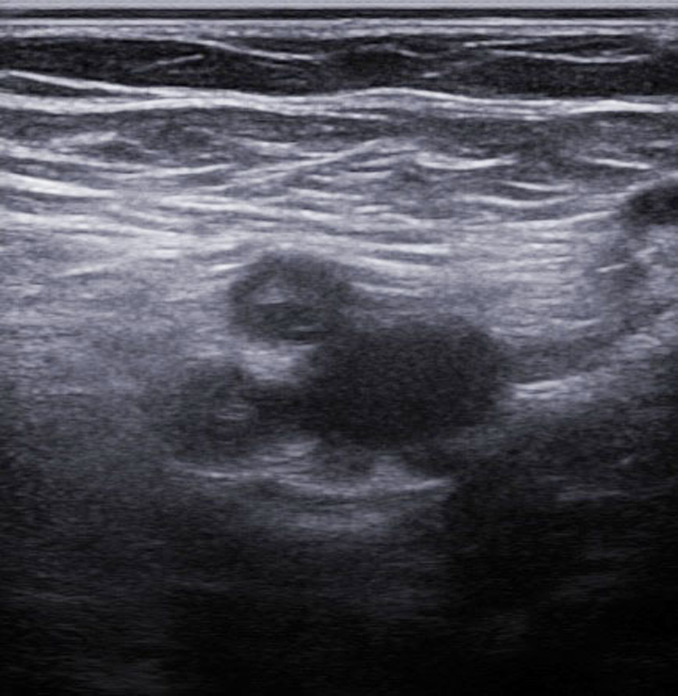
echo Doppler of left lower limb showing occlusion of femoral axes

**Figure 5:**
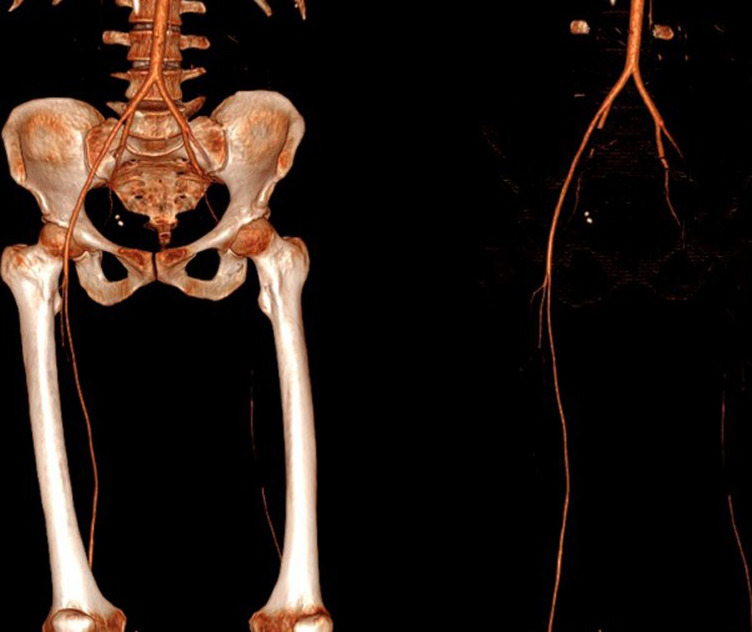
angio-CT scan of lower limb showing an interruption of vascular bed at the level of common femoral artery with partial resumption in the popliteal artery

Transthoracic Echocardiography (TTE) showed a biventricular dilated cardiomyopathy (DCM), severe biventricular dysfunction (LV ejection fraction (LVEF): 10%), low cardiac index at 1.20 L/min/m^3^ with a large intra LV “horseshoe” thrombus, showing also a high heart LV filling pressure with elevated PAPs at 51 mmHg and a dilated inferior vena cava (Figure 2, Figure 3). US troponin and ProBNP were very high at 734 ng/l and 2215 pg/ml respectively. Echo Doppler of lower limbs showed a right popliteal vein thrombosis and an occlusion of femoral axes in the left lower limb (Figure 4). A complement by an angio-CT scan was performed which confirmed the arterial occlusion of femoral axes (Figure 5).

**Figure 6:**
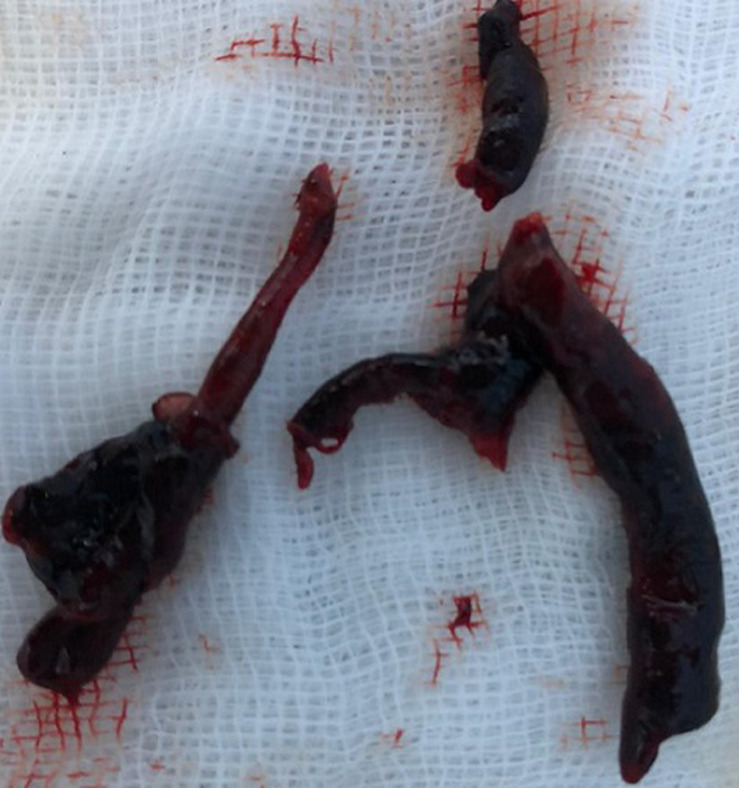
post-operative image showing the thrombi after FOGARTY of left lower limb

Therapeutic strategy chosen was to put the patient on vasoactive drugs (dobutamine and norepinephrine), and after hemodynamic stabilization, we have introduced the treatment of heart failure: Bisoprolol, Ramipril, Spironolactone and Furosemide. An embolectomy using the FOGARTY probe was performed for the left lower limb (Figure 6). For anticoagulation, she was initially on curative dose of Low Molecular Weight Heparin (LMWH), then relayed with Acenocoumarol associated with antiplatelet therapy by aspirin and topically applied heparin. For COVID-19 infection, she was treated by antibiotic coverage (ceftriaxone 2 g/d, ciprofloxacin 1 g/d), methylprednisolone 32 mg/d and vitamin supplementation. The patient remained hemodynamically stable (BP: 120/75 mmHg, HB: 80 bpm) with improvement in respiratory function (SpO2: 94% in ambient air) and resumption of left limb pulses. After an incident free follow up of thirty days, the TTE showed a slight improvement in systolic function (LVEF at 30%), with a clear regression of the intra LV thrombus, the right ventricle has well recovered its function.

## Discussion

Extra-respiratory COVID-19 complications including cardiac injuries have been frequently reported for being associated with increased mortality. According to the literature, cardiac injury is defined as the increasing of troponin and/or natriuretic peptides levels [2]. Among these complications, there are myocardial injuries that may progress to systolic dysfunction and arteriovenous thromboembolic complications, which are frequently reported, especially in hospitalized patients. In two Chinese cohorts, a prevalence of myocarditis during COVID-19 was described in approximately 19.7% to 27.8% of cases [2, 3]. In addition, Klok *et al*. have showed an incidence of venous thromboembolism at 27%; whereas that in arterial events was 3.7% [1]. These figures may decrease over time due to renewable therapeutic strategies in this domain.

In myocardial injury, cell lysis caused by direct viral toxicity and inflammatory phenomena can induce the activation of the inflammatory response (pro-inflammatory cytokines), which are responsible for the destruction of cardiomyocytes, and then ventricular dysfunction that can evolve into cardiogenic shock. Endothelial cell infection has also an important place in disease chronization and later dilated cardiomyopathy with severe ventricular dysfunction [4]. It is conventional to introduce a preventive dose of anticoagulation in hospitalized COVID-19 patients, but despite that, several cases had developed thromboembolic complications that lead towards death. Pathophysiology is not yet well explained, but most studies have found a state of hypercoagulability in these patients (high level of D-dimer, fibrinogen, Factor VIII, Antithrombin, Antiphospholipid antibodies with decreased protein C, protein S). On another side, there is Virchow's triad (stasis related to bed rest and obesity, parietal damage due to endothelial inflammation, intra ventricular stasis and hypercoagulability due to sepsis), which are already common in COVID-19 [5]. In our case, thrombus constitution was due to intra LV stasis and LV dysfunction, hypercoagulability was due to COVID-19 infection and endothelial lesions. Thus, lower limb ischemia was due to the immigration of intra LV thrombus. Because of its severity, myocarditis should be investigated in patients with COVID-19, who present with warning signs such as low blood pressure, tachycardia or signs of heart failure. TTE and dosage of cardiac enzymes (Troponin and BNP) are essential for making the diagnosis. Cardiac magnetic resonance imaging (MRI) is not routinely used for the diagnosis of COVID-19 myocarditis due to the technical difficulty [4]. Doppler Ultrasound of lower limbs is a principal element for the diagnosis of venous thrombosis and arterial ischemia. Angio-CT allows a better characterization of thrombosis and guides the therapeutic strategy.

Regarding management of myocardial injuries during COVID-19, Renin-angiotensin-aldosterone system (RAAS) inhibitors and beta-blockers should be maintained [6]. Corticosteroids, sometimes with immunoglobulins, have been administered in cases of suspected severe myocarditis and in a context of severe inflammation. In the case of fulminant myocarditis, vasoactive drugs and mechanical circulatory support have their place [7]. With regard to anticoagulation management, The American College of Chest Physicians (ACCP) suggests a prophylaxis by LMWH or fondaparinux for hospitalized patients in the absence of contraindications [8]. Whereas, the International Society on Thrombosis and Hemostasis (ISTH) suggests a half dose of LMWH as prophylaxis for high-risk patients, with a 50% higher dose for whom with obesity [9]. In addition, the ACCP recommends a prophylactic anticoagulation only during hospitalization [8], while the ISTH recommends it even at discharge in patients with a low risk of bleeding, with a duration of 14 to 30 days [9]. High-risk features in COVID-19 include older than 65 years, critical illness, cancer, prior VTE, thrombophilia, severe immobility, and elevated D-dimer (>2 times the upper limit of normal) [9]. It is conventional to put these thromboembolic complicated patients on well-managed and well-monitored curative anticoagulation. Some studies have shown a platelet hyperactivity in patients with COVID-19, and that paves the way to discuss the benefit of antiplatelet therapy [10].

Despite everything, the best management of these complications are still being discussed, and while waiting for other studies that will show more efficiency, we should follow current guidelines towards them to avoid as much as possible the aggravation of these cases, which may lead to a high mortality rate.

## Conclusion

SARS-CoV-2 infection has been associated with myocardial injuries and a hyper coagulable state that can be responsible of thromboembolic complications, which have a high risk of mortality, especially in critically ill patients. In the absence of contraindications, thromboprophylaxis should be initiated for all hospitalized patients with COVID-19. Whilst waiting for other studies, treatment of myocarditis COVID-19 involves the treatment of heart failure, corticosteroids, sometimes with immunoglobulins.
